# Editorial: Developmental Disorders of the Kidney and Urinary Tract: Recent Insights From Clinical and Molecular Studies

**DOI:** 10.3389/fped.2020.00348

**Published:** 2020-06-26

**Authors:** Eduardo A. Oliveira, Robert H. Mak, Ana Cristina Simões e Silva

**Affiliations:** ^1^Pediatric Nephrology Unit, Department of Pediatrics, Federal University of Minas Gerais, Belo Horizonte, Brazil; ^2^Division of Pediatric Nephrology, Rady Children's Hospital San Diego, University of California, San Diego, La Jolla, CA, United States; ^3^National Institute of Science and Technology (INCT) of Molecular Medicine, Belo Horizonte, Brazil

**Keywords:** congenital anomalies of the kidney and urinary tract, chronic kidney disease, prenatal diagnosis, fetal hydronephrosis, renal hypodysplasia, genetics, molecular mechanisms, gene polymorphism

Outstanding advances have been obtained in basic and clinical research on congenital anomalies of the kidney and urinary tract (CAKUT) over the past decade. From the molecular point of view, new generation sequencing has made crucial contributions to our understanding of the biology and pathophysiology of disrupted renal development, including the identification of associated genes and insights into the cellular pathophysiology (1, 2). Approximately 40 different monogenic causes for human CAKUT have so far been identified. Nevertheless, at present only about 20% of CAKUT cases can be explained by these established monogenic causes (3–5). Therefore, it is probably that several additional monogenic causes of human CAKUT have yet to be identified. In this Research Topic, Woolf et al. have demonstrated several rare diseases among the CAKUT complex with defined genetic causes. These studies are indicating that the implicated genes encode smooth muscle, neural or urothelial molecules, or master transcription factors that regulate their expression. However, variants in these same genes do not appear to explain the more common human non-syndromic urinary tract malformations such as primary vesicoureteral reflux. Of note, studies with whole exome sequencing, a technology that seeks variants in the protein coding regions of all genes, is being applied to seek likely pathogenic mutation in clinical cohorts of children born with a range of kidney malformations (6). Such researches have yielded useful genetic information in 10–14% of cases tested (Woolf et al.). Taroni et al. reported, on this Research topic, a case of a 2 years old child with Hypotonia-Cystinuria syndrome (HCS). HCS is a rare disease, caused by a mutation in two contiguous genes (SLC3A1 and PREPL), localized on chromosome 2p21, and it is characterized by both renal involvement with cystine stones and nervous involvement with hypotonia. Interestingly, the case reported had HCS associated with other clinical features of CAKUT, primary obstructed megaureter (POM), cryptorchidism and cardiac involvement. Some clinical features showed in this case report, like cryptorchidism and POM, have never been reported before in patients with HCS.

From the clinical point of the view, CAKUT encompass a wide range of structural malformations, including a complex spectrum of abnormalities that occur at the level of the kidney (e.g., hypoplasia and dysplasia), ureter (e.g., hydronephrosis and megaureter), bladder (e.g., ureterocele and vesicoureteral reflux), and urethra (e.g., posterior urethral valves) (7). CAKUT are estimated to be implicated in 30–60% of cases of childhood-onset chronic kidney disease (CKD) in different populations (8–10). CAKUT are a heterogeneous and complex group of congenital disorders which can lead to long-term health risks of hypertension, recurrent urinary tract infection (UTI), CKD, and end stage renal disease (ESRD) in children (11–14).

Due to the recognized role played by CAKUT as primary causes in a sizeable proportion of pediatric CKD, it is of crucial importance that early diagnosis and tailored management are enacted to minimize renal damage and especially with respect to prevent or delay the onset of CKD. In this regard, over the last decade, pediatric disciplines have been markedly influenced by fetal screening ultrasonography, notably pediatric nephrology, and urology. An increasing number of renal anomalies are currently detected at antenatal stage in otherwise uncomplicated pregnancies. As a matter of fact, the majority of CAKUT is currently detected by antenatal ultrasonography; although still many cases remain undiagnosed until adulthood. Prenatal detection has permitted a refinement of the management of these conditions. A multidisciplinary team approach, including obstetrics, neonatology, pediatric nephrology, and pediatric urology is clearly required to diagnose and treat these complex disorders (15, 16). Consistent clinical information regarding diagnosis and prognosis should be provided during pregnancy and after birth. The calculated prognosis regarding the severity of the disease, survival and quality of life will have a major impact on decision-making during both pregnancy and postnatal period. In this regard, Katsoufis et al. presented on this Research Topic, a cohort of 42 subjects followed prospectively from birth for an average of 6.1 ± 2.8 years. The authors showed that a neonatal CysC >3.0 mg/L predicted progression to ESRD, while a nadir serum creatine >0.6 mg/dL predicted progression to CKD 3–5 with the highest specificity and sensitivity by ROC-AUC analysis (*P* < 0.0001). Interestingly, Vasconcelos et al. (14) developed a clinical predictive model to assess individualized risk of post-natal surgical intervention in patients with antenatal detected CAKUT. Predictors of the surgical intervention in the model were: baseline glomerular filtration rate, associated hydronephrosis, presence of renal damage and the severity of renal pelvic dilatation. Concerning the selection of infants with CAKUT for surgical intervention, Damasio et al. compare functional magnetic resonance (MR) urography with dynamic renal (DRS) scintigraphy in measuring volumetric split renal function (SRF) and in the classification of drainage curves. The authors showed that there were no significant differences between functional MR urography and DRS in measuring volumetric SRF and in the classification of drainage curves in patients with CAKUT.

The long-term impact of the earlier diagnosis and management of CAKUT needs to be addressed by longitudinal studies, including significant clinical outcomes, such as proteinuria, hypertension, and renal survival. In this regard, Chiodini et al. presented on this Research Topic, a comprehensive review of the clinical outcomes of the most frequently discovered by post-natal screening, including uretero-pelvic junction stenosis (UPJS), primary vesicoureteral reflux (VUR), megaureter, duplex kidney, and posterior urethral valves (PUV). Interestingly, Gabriele and Koch Nogueira have shown that hypertension is an independent risk factor for kidney disease progression and should be promptly managed for renal protection, especially among patients with CAKUT, the most common cause of CKD in the pediatric population.

In summary, CAKUT are responsible for 20–30% of all prenatally detected anomalies and are the most common cause of CKD in infants. A multidisciplinary team approach is optimal to diagnose and manage these complex disorders. The long-term impact of the data-based management of these renal abnormalities needs to be addressed by future studies, including significant clinical outcomes, such as proteinuria, hypertension, and CKD. Advances in prenatal diagnosis, genetic testing, fetal surgery, organ transplantation, and surgical treatment of CAKUT have improved the prognosis and quality of life of affected patients. CAKUT have significant impact in clinical medicine and across various subspecialties. Regardless of current difficulties and limitations, molecular genetic diagnoses may offer hopeful advances to the clinical management of patients with CAKUT. For instance, identification of causative mutations perhaps will contribute to relevant aspects of pediatric care including prognostic prediction of renal survival and the likelihood for the development of extrarenal complications. Nevertheless, in spite of this outstanding progress, many issues remain to be solved in order to integrate these approaches into clinical practice.

## Author Contributions

EO, RM, and AS: conceptualization and writing—review and editing. EO: writing—original draft preparation. All authors contributed to the article and approved the submitted version.

## Conflict of Interest

The authors declare that the research was conducted in the absence of any commercial or financial relationships that could be construed as a potential conflict of interest.
